# Biochemical and molecular characterization of three serologically different *Vibrio harveyi* strains isolated from farmed *Dicentrarchus labrax* from the Adriatic Sea

**DOI:** 10.1038/s41598-022-10720-z

**Published:** 2022-05-04

**Authors:** Željko Pavlinec, Ivana Giovanna Zupičić, Dražen Oraić, Ivana Lojkić, Belén Fouz, Snježana Zrnčić

**Affiliations:** 1 Croatian Veterinary Institute, 10000 Zagreb, Croatia; 2 University of Valencia, 46010 Valencia, Spain

**Keywords:** Microbiology, Molecular biology, Diseases

## Abstract

*Vibrio harveyi* is recognized as one of the major causes of vibriosis, a disease that threatens the long-term sustainability of aquaculture. Current research shows that the Mediterranean strains of *V*. *harveyi* are serologically heterogeneous, though research comparing the traits of different strains is scarce. This study aims to describe the biochemical, physiological and genetic characteristics of three serologically different strains of *V*. *harveyi* isolated from farmed European Sea bass (*Dicentrarchus labrax*) from the Adriatic Sea. A total of 32 morphological and biochemical markers were examined and, the susceptibility to 13 antimicrobials tested, and then compared the results of high-throughput sequencing and in silico analyses. This study also presents the first whole genome sequences of *V*. *harveyi* isolated from European sea bass. A large number of nonsynonymous variations were detected among sequences of the three strains. The prediction analysis of resistance genes did not correspond with the in vitro antimicrobial susceptibility tests. Six virulence genes previously unrelated to virulence of vibrios were detected in all three studied strains. The results show that differences were detected at every level of comparison among the three studied strains isolated from the same fish species originating from a small geographic area.

## Introduction

*Vibrio harveyi* is a Gram-negative, luminous, halophilic bacteria belonging to the family *Vibrionaceae*. Initially, it was considered apathogenic^1^, until it was discovered to be the cause of vasculitis and dermal lesions and cysts in sandbar shark (*Carcharhinus plumbeus*)^2^. Since then, it has been globally described as a pathogen of many marine animals, such as common snook (*Centropomus undecimalis*) in the Gulf of Mexico^3^, shrimps *Penaeus monodon* and *P*. *merguiensis* in Indonesia, Thailand and Philippines^4^, silvery black porgy (*Sparidentex hasta*) and brown-spotted grouper (*Epinephelus tauvina*) in the Persian Gulf^5^, common dentex (*Dentex dentex*), gilthead sea bream (*Sparus aurata*) and European sea bass (ESB) (*Dicentrarchus labrax*) in the Balearic Sea^6,7^, sea cucumber (*Holothuria scabra*) in the Coral Sea^8^ and the south-west coast of Madagascar^9^, rock lobster (*Jasus verreauxi*) in Mahanga Bay, New Zealand^10^, common seahorse (*Hippocampus kuda*) in Tenerife, Spain^11^, Senegalese sole (*Solea senegalensis*) in the Alboran Sea^12^, tropical stony corals in Indonesia^13^, Asian sea bass (*Lates calcarifer*) in Philippines and Malaysia^14,15^, greater amberjack (*Seriola dumerili*) in the Mediterranean Sea^16^ and hybrid grouper (*Epinephelus fuscoguttatus* × *Epinephelus lanceolatus*) in the Yellow Sea^17^.

In the last two decades, *V*. *harveyi* has been recognized as one of the major causes of vibriosis^18–21^, a disease that causes severe losses in aquaculture and threatens its long-term sustainability. Many examples found in the literature are as follows. High mortalities, even up to 100%, due to vibriosis caused by *V*. *harveyi* have been reported in several species of molluscs, crustaceans and fish^19^. In 2010, two major disease outbreaks in different marine fish species farmed in Sulaman Bay, Malaysia, were caused by *V*. *harveyi*^22^. Shen et al. reported mortalities of up to 90% of cultured juvenile hybrid groupers in 2017 when the disease was not treated in time^17^. Mortalities of up to 40% of farmed Asian sea bass have been reported from Vietnam^23^. In 2019, Norhariani et al. estimated the average cost of vibriosis to 0.24 USD per tail for Asian sea bass cage culture^24^. Vibriosis has caused major production declines in the prawn industry, and Asian aquaculture has estimated losses of 1 billion USD due to vibriosis of shrimps^25^. In the Mediterranean, aquaculture disease outbreaks due to *V*. *harveyi* are becoming more common in the last decade. Outputs of the EU Horizon 2020 project “MedAID” summarized the mortalities in Mediterranean marine aquaculture caused by bacterial diseases and emphasized the frequent occurrence of vibriosis for both prevalent fish species, European sea bass and gilthead sea bream^26^.

Apart from mortalities, there are other economic consequences of vibriosis. Major economic impacts and increased losses come from slow larval metamorphosis, retarded growth and body malformations^27^, reduced price due to visible signs of disease, international trade restrictions of diseased animals and, consequently, the reduced job security of workers in aquaculture^25^.

Regarding virulence, *V*. *harveyi* strains are highly diverse, with some causing high mortalities while others are non-virulent^18,28^ and research efforts into its virulence genes were employed to clarify the pathogenicity of this bacterium. It was reported that the main virulence factors of *V*. *harveyi* are extracellular products (ECPs)^28,29^, outer membrane proteins^20^, quorum sensing^30–33^, secretion system^32^ and motility^33^. Major virulence factors of the best-characterized bacterial pathogens, including several *Vibrio* species, are covered in the virulence factor database (VFDB)^34^.

*Vibrio harveyi* became an emerging pathogen in the Mediterranean marine aquaculture causing economic losses during last ten years mostly due to climate changes. Until recently, most studies on the *V. haveyi* were published from subtropical, mostly South Eastern Asian aquaculture and there are several reports from Mediterranean region^21^.

Using serological techniques originally developed for the characterization of *V*. *anguillarum*^35^ and *V*. *vulnificus*^36^, it was demonstrated that strains of *V*. *harveyi* isolated from diseased farmed ESB along the Iberian Peninsula coast are not homogeneous and that serotype A seems to be dominant, especially among the most virulent strains^37^. According to the latest results, there appear to be three dominant serotypes among the Mediterranean *V*. *harveyi* strains^21^. Currently, the research comparing the characteristics of different serotypes of *V*. *harveyi* is scarce.In recent years, outbreaks of vibriosis in ESB, associated with *V*. *harveyi*, have been recorded in the summer months at several farms along the Croatian Adriatic coast. The bacteria is normal inhabitant of the marine environment^38^ and our main interest was to compare isolates belonging to intestine microbiome of clinically healthy fish and strain isolated from clinically diseased ESB with typical symptoms for infection with *V. harveyi* belonging to three different serotypes. To authors’ knowledge this is the first whole genome sequencing of *V. harveyi* strains isolated from Mediterranean ESB, more precisely from the Adriatic Sea.

## Results

### Biochemical and serological characteristics of *V. harveyi*

Strains 72/16, 150/16 and 94/17 were confirmed as *V*. *harveyi* by using Nucleotide BLAST against NCBI Nucleotide database with the obtained 16S rRNA and *toxR* sequences. Strains 72/16 and 150/16 were part of the intestine microbiome collected from farmed ESB during winter months, while strain 94/17 was isolated from ESB with typical symptoms during August. Serological assays showed that strain 94/17 belongs to serotype A and strain 150/16 belongs to the new serotype recently reported in Croatian isolates (tentatively, serotype B), while strain 72/16 is serologically different from those belonging to the known serotypes. The biochemical and physiological characteristics of the studied strains compared with reference characteristics published by Austin & Austin^39^ and Pretto^40^ are presented in Table 1. Of the 32 tested characteristics, all three strains were identical for 25 and differed in seven traits. All three strains are gram-negative, oxidase and catalase positive, capable of fermenting saccharose, motile and grow as yellow colonies on TCBS agar. They are all positive for lysine decarboxylase and can utilise citrate, but are negative for β-galactosidase, arginine dihydrolase, urease, acetoin and production of hydrogen sulphide. Furthermore, all three strains can ferment glucose, mannose, sucrose and amygdalin while none can ferment inositol, rhamnose, melibiose or arabinose. Regarding the salt tolerance, the strains can all grow in peptone water with up to 6% added salt but are unable to grow in peptone water with 10% salt. Regarding their differences, strains 94/17 and 150/16 are α-haemolytic while strain 72/16 is β-haemolytic. Likewise, strains 94/17 and 150/16 are positive for ornithine decarboxylase and tryptophanase and can ferment sorbitol, while strain 72/16 does not possess those traits. For tryptophan deaminase, strains 94/17 and 72/16 are positive while strain 150/16 is negative. The reverse is true for gelatinase, with only strain 150/16 positive. Finally, strain 94/17 is the only one able to reduce nitrates to nitrites same as reference strains^39,40^. Compared to previously published characteristic, most of the properties are the same excluding the fact that none of Croatian studied strains fermented arabinose.

**Table 1:** Biochemical and physiological properties of *V*. *harveyi* strains.

Strain	94/17	72/16	150/16	*V. harveyi* (Austin and Austin, 2007; Pretto, 2020)
Gram staining	−	−	−	−
Oxidase	+	+	+	+
Catalase	+	+	+	+
OF glucose	F	F	F	F
TCBS	Yellow	Yellow	Yellow	Yellow
Motility	+	+	+	+
Type of haemolysis	α	β	α	α or β
ONPG	−	−	−	−
ADH	−	−	−	−
LDC	+	+	+	+
ODC	+	−	+	+
CIT	+	+	+	+
H_2_S	−	−	−	−
URE	−	−	−	−
TDA	+	+	−	−
IND	+	−	+	+
VP	−	−	−	−
GEL	−	−	+	+
GLU	+	+	+	+
MAN	+	+	+	+
INO	−	−	−	−
SOR	+	−	+	+
RHA	−	−	−	−
SUC	+	+	+	+
MEL	−	−	−	−
AMY	+	+	+	+
ARA	−	−	−	+
NO_2_	+	−	−	+
NaCl 0.5%	+	+	+	+
NaCl 3%	+	+	+	+
NaCl 6%	+	+	+	+
NaCl 10%	−	−	−	−

OF = glucose oxidation-fermentation test, F = fermentative, ONPG = β-galactosidase, ADH = arginine dihydrolase, LDC = lysine decarboxylase, ODC = ornithine decarboxylase, CIT = citrate, H_2_S = hydrogen sulphide production, URE = urease, TDA = tryptophan deaminase, IND = indole, VP = Voges-Proskauer, GEL = gelatinase, GLU = glucose, MAN = mannitol, INO = inositol, SOR = sorbitol, RHA = rhamnose, SUC = sucrose, MEL = melibiose, AMY = amygdalin, ARA = arabinose, NO_2_ = reduction of nitrates to nitrites.

### Susceptibility of *V, harveyi* to different antimicrobials

The results of the antimicrobial susceptibility test are presented in Table 2 as the measured diameters of inhibition zones obtained by the disc diffusion method since there is no harmonised interpretive criteria for *V. harveyi*^41^ Obtained results showed that none of the tested strains inhibited growth of ampicillin, O129 10 μg and Novobiocin where only strain 72/16 showed slight inhibition. Lower inhibition was obtained for Gentamicin for three strains while there are smaller zones of inhibition for all substances of the strain 72/16 compared to other two tested strains.

**Table 2:** Susceptibility of *V*. *harveyi* strains to antibacterial substances.

Antibiotic	Bacterial strain		
	94/17	72/16	150/16
Ampicillin 10 µg	0*	0	0
Ceftazidime 30 µg	53	37	46
Chloramphenicol 30 µg	43	40	47
Enrofloxacin 5 µg	53	34	47
Florfenicol 30 µg	50	39	45
Gentamicin 10 µg	25	20	22
Meropenem 10 µg	55	40	53
Novobiocin 5 μg	0	9	0
O129 10 μg	0	0	0
Oxolinic acid 2 µg	40	28	42
Oxytetracycline 30 µg	40	31	39
Sulfamethoxazol 300 µg	45	32	38
Trimethoprim / Sulfamethoxazole 1.25/23.75 µg	33	31	40

*Numbers represent the diameter of the inhibition zones in mm.

### Sequencing results

Sequencing on the MiSeq system produced 1,483,562 reads totalling 315,351,026 nucleotides for strain 94/17, 2,395,144 reads totalling 459,482,093 nucleotides for strain 150/16 and 1,639,392 reads totalling 334,434,520 nucleotides for strain 72/16. Data obtained by sequencing is available in the SRA database^42^ with the accession numbers SAMN18310299, SAMN18310300, SAMN18310301 for strains 94/17, 72/16 and 150/16, respectively.

**Figure 1:**
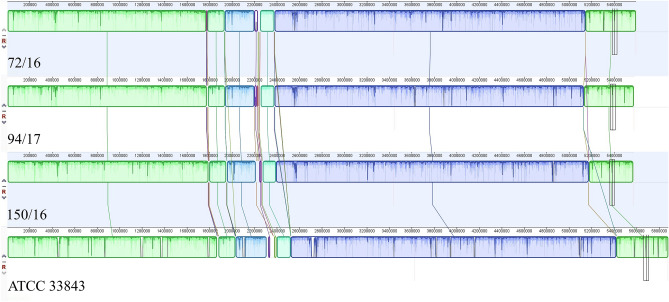
Progressive Mauve alignment of the consensus whole genome sequences of *V*. *harveyi* strains 72/16, 94/17, 150/16 and ATCC 33,843. The homologous regions are represented as collinear blocks with the same colour.

Different parameters describing the consensus sequences obtained by mapping are presented in Table 3. Mean coverage is from 42.9 for chromosome 1 of strain 94/17 to 67.2 for chromosome 1 of strain 150/16. Total length is shortest for strain 150/16 with 5,571,304 base pairs (bp), followed by strain 94/17 with 5,578,489 bp, and the longest in strain 72/16, with 5,594,934 bp. With relation to the reference sequence (ATCC 33,843) the mapping of strain 94/17 covered 94.8%, strain 72/16 covered 95.1% and mapping the strain 150/16 covered 94.7% of the genome. GC-content is similar in all three strains, with 45.12% in strain 94/17, 45.14% in strain 72/16 and 45.13% in strain 150/16. Strain 94/17 is the most similar strain to the reference, with 98.7% pairwise identity, followed by strain 150/16 with 98.1% pairwise identity. The difference is slightly higher in strain 72/16 with 97% pairwise identity with the reference strain. The number of transferred annotations is similar in all three strains with 4893, 4913 and 4895 annotated genes, in strains 94/17, 72/16 and 150/16, respectively. The whole genome alignment is presented in Fig. 1.

**Table 3:** Parameters of the consensus whole genome sequences.

Name	No. of mapped reads^a^	Mean coverage^a^	Length (bp)	GC-content (%)	Reference covered^b^ (%)	No. of annotated genes
94/17 chromosome 1	728,469	42.9	3,444,186	45.2	95.1	3,050
94/17 chromosome 2	468,158	44.2	2,134,303	45.0	94.4	1,843
72/16 chromosome 1	870,861	49.4	3,440,054	45.2	95.0	3,042
72/16 chromosome 2	502,691	45.5	2,154,880	45.0	95.4	1,871
150/16 chromosome 1	1,208,581	64.3	3,438,391	45.2	94.9	3,029
150/16 chromosome 2	711,520	60.6	2,132,913	45.0	94.4	1,866

^a,b^In relation to *V. harveyi* strain ATCC 33,843.

The total number of nonsynonymous variants in nucleotide sequences between strains 72/16 and 94/17 is 9462 with 5184 on chromosome 1 and 4,278 on chromosome 2. Of those, there are: 93 deletions, 72 insertions, 7,101 single nucleotide polymorphisms (SNPs) and 2196 substitutions, resulting in following protein changes: 39 deletions, 44 extensions, 82 frame shifts, 23 insertions, 12 start codon losses, 9151 substitutions and 111 truncations. A comparison of strain 150/16 with strain 94/17 found a total of 11,280 nonsynonymous variants in nucleotide sequences, 6574 on chromosome 1 and 4706 on chromosome 2. Based on the polymorphism type they are divided into 125 deletions, 126 insertions, 7695 SNPs and 3,334 substitutions, while based on their effect on the protein into 40 deletions, 57 insertions, 144 frame shifts, 34 insertions, 18 start codon losses, 10,764 substitutions and 223 truncations. Finally, between the strains 150/16 and 72/16, the total number of nonsynonymous variants in nucleotide sequences is 10,952 with 5989 on chromosome 1 and 4963 on chromosome 2. Here, they are divided based on the polymorphism type into 125 deletions, 120 insertions, 7924 SNPs and 2783 substitutions, and based on the protein change into 35 deletions, 140 extensions, 159 frame shifts, 30 insertions, 18 start codon losses, 10,359 substitutions and 211 truncations.

Using Resistance Gene Identifier (RGI) analysis in the Comprehensive Antibiotic Resistance Database (CARD)^43^ we identified the same 374 genes linked to antimicrobial resistance (AMR) in each of the reference mapped consensus genomes of the studied strains. Of those, there were zero perfect hits, four strict hits and 370 loose hits. The four strict hits were the same in every strain, and are presented in Table 4. All hits are shown in Supplementary Table S1.

**Table 4:** Strict hits obtained by RGI analysis in CARD.

ARO term	AMR gene family	Drug class	Resistance mechanism
tet(35)	ATP-binding cassette (ABC) antibiotic efflux pump	Tetracycline antibiotic	Antibiotic efflux
CRP	Resistance-nodulation-cell division (RND) antibiotic efflux pump	Macrolide antibiotic, fluoroquinolone antibiotic, penam	Antibiotic efflux
adeF	RND antibiotic efflux pump	Fluoroquinolone antibiotic, tetracycline antibiotic	Antibiotic efflux
*Escherichia coli* parE conferring resistance to fluoroquinolones	Fluoroquinolone resistant parE	Fluoroquinolone antibiotic	Antibiotic target alteration

ARO = antibiotic resistance ontology; AMR = Antimicrobial resistance.

Using the VFanalyzer, we identified 155 genes associated with virulence. They can be divided, according to function, into 55 genes related to mobility, 48 genes related to secretion, 19 genes related to adherence, 16 genes related to antiphagocytosis, 11 genes related to iron uptake, two genes related to quorum sensing, two genes related to toxins, one gene related to cell surface components and one gene related to fimbrial adherence. The entire result of the analysis is presented in Supplementary Table S2. Table 5 singles out the genes found in this analysis, but not in all three studied strains, and also shows the genes that were not associated with virulence of other *Vibrio* species in the VFDB. In total, seven virulence genes are identified only in one or two of the studied strains, and there are eight genes previously not linked with virulence in other *Vibrio* species.

**Table 5:**
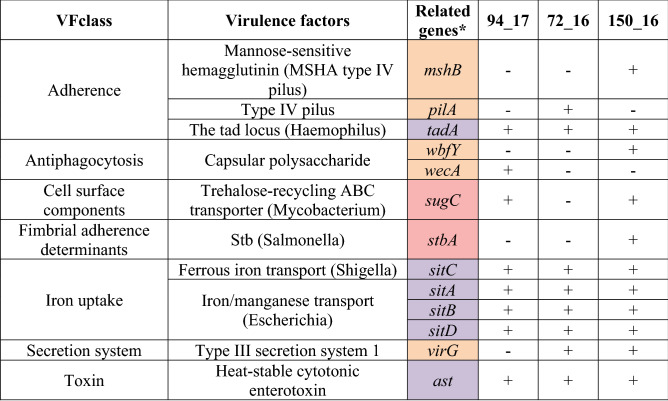
Virulence genes detected by VFanalyzer with differences among *V*. *harveyi* strains or associated with the genus *Vibrio* for the first time.

*Orange—virulence genes with differences among the studied strains; purple—virulence genes associated with genus *Vibrio* for the first time; red—virulence genes associated with genus *Vibrio* for the first time with differences among the studied strains.

## Discussion

*V*. *harveyi* is one of the major species responsible for vibriosis, a disease globally causing severe economic losses in aquaculture. Still, the exact mechanism and interplay of different factors underlying this disease are largely unclear, and different strains of *V*. *harveyi* show large phenotypic differences in regards to their virulence^18,28,30^. This study describes the first approach to comparing the biochemical, physiological and genetic characteristics of serologically different *V*. *harveyi* strains originating from the Mediterranean Sea. The comparison includes two strains belonging to identified *V*. *harveyi* serotypes (A and tentatively “B”) and a strain serologically different from those.

Comparing the biochemical characteristics of the studied strains obtained by the API 20E test, we noted that all three strains are similar with 15 of 21 reactions giving the same result. Still, certain unexpected differences were obtained, with strain 72/16 negative for ornithine decarboxylase and tryptophanase, strains 72/16 and 150/16 negative for the reduction of nitrates and strains 94/17 and 72/16 negative for gelatinase. Positive results were expected for these traits, as reported by Austin and Austin^39^. With two more reactions showing dissimilar results, tryptophan deaminase and sorbitol tests, there is clearly some ambiguity between *V*. *harveyi* strains, and therefore we advise caution in solely relying on this analysis for identification. Further, strain 72/16, which is serologically different from previously identified serotypes, deviates even further from expectations and is more diverse from the strains of the known serotypes than they are in relation to each other.

To date, the zone diameter clinical breakpoints of the disc diffusion test are published for only one aquatic bacteria, *A*. *salmonicida*^41^, and have yet to be defined based on standardised testing protocol and harmonised interpretive criteria in susceptibility studies for *V*. *harveyi*^44^. For this reason, the results of antimicrobial susceptibility are presented as measured diameters of inhibition zones without interpretation as to whether the bacteria are sensitive or resistant to a given antibiotic. Still, it appears that the studied bacterial strains are inhibited by all tested antimicrobials except ampicillin, novobiocin and the 10 μg disc of vibriostat O129. Comparing the results of the rest of the antimicrobials, strain 72/16 shows smaller inhibition zones than strains 94/17 and 150/16 in each test, as seen by the average size of inhibition zones for each strain (strain 94/17: 43.7 ± 9.6; strain 150/16: 41.9 ± 8.3; and strain 72/16: 33.2 ± 6.3) across all tested antimicrobials demonstrating the inhibition of growth. The strains belonging to serotypes A and “B” show highly similar zones of inhibition.

In terms of the consensus genome sequences, it appears that some genomic data are missing in all three strains. Compared to the reference genome, which comes from one of the most studied strains (ATCC 33,843)^45^, the consensus sequences of all three of our studied strains are shorter by 0.3 Mb, with over 400 fewer gene annotations. This is most easily observed using the whole genome alignment (Fig. 1), indicating that a part of the reference genome is not present in the studied strains. Meanwhile, the number of unmapped reads is relatively high for all three strains (strain 94/17: 19.34%, strain 72/16: 16.22%; strain 150/16: 19.83%). This indicates that a large amount of sequenced DNA found in the strains from the Adriatic Sea is not present in the reference strain. While the existence of large plasmids in *V*. *harveyi* has been demonstrated^46,47^, considering the percentage of unmapped reads it is unlikely that they all belong to plasmid DNA. Comparing the whole genome alignment of the reference mapped consensus sequences of the studied strains, several areas can be observed with a higher concentration of variation, and there are some smaller differences in size between the collinear blocks. There is a large number of nonsynonymous variations between the three studied strains and it would be interesting to explore whether the same mutations are found in other strains belonging to the same serotypes, linking exact variations to functional diversity.

The RGI analysis using strict criteria predicted resistance to four drug classes in all three strains. All four genes can be found in CARD associated to antimicrobial resistance in different *Vibrio* species, but to our knowledge, only *tet35* has previously been associated with *V*. *harveyi*^48^. Comparing the results of the RGI analysis with the disk diffusion test of antimicrobial sensitivity, several observations can be made. The only in silico predicted resistance that manifested in vitro is for ampicillin (penam). While the RGI analysis predicted resistance to tetracycline antibiotics and fluoroquinolone antibiotics, this was not confirmed with disc diffusion. Quite the opposite, the inhibition zones were from 31 to 40 mm for oxytetracycline disks, and 34 to 53 mm for enrofloxacin disks. Finally, the in vitro analysis showed that the strains were resistant to novobiocin, an aminocoumarin antibiotic, and while the RGI analysis identified 28 genes linked to aminocoumarin antibiotic resistance, all were loose hits. Considering these results, we can conclude that the results of the RGI analysis should be considered with caution and they clearly cannot replace the in vitro assay. Unfortunately, analysing RGI in CARD resistance genes associated to the reference strain of *V. harveyi* ATTCC 33,843 showed single *adeF* gene what additionaly emphasize the need of more comprehensive research with inclusion of more isolates of this important species of the genus *Vibrio*.

Currently, there are 242 genes in the VFDB associated with virulence of at least one of the following *Vibrio* species: *V*. *cholerae*, *V*. *fischeri*, *V*. *parahaemolyticus* and *V*. *vulnificus*. Of those 242 genes, 142 are found in all three of the studied *V*. *harveyi* strains, while five other genes were found in at least one of the studied strains. Additionally, eight virulence genes were detected that were previously unrelated to the virulence of vibrios, and six of those eight genes were detected in all three of our studied strains. Five genes were associated with only one strain of *V*. *harveyi* (strain 94/17: *wecA*; strain 72/16: *pilA*; and strain 150/16: *mshB*, *wbfY* and *stbA*). Future research should examine whether these genes are always found in specific serotypes.

In conclusion, we found differences at every level of comparison among the three studied *V*. *harveyi* strains isolated from the same fish species originating from a small geographic area. We should keep in mind that studied strains were isolated in different environmental condition what could influence on the expression of the virulence and resistance genes. However, neither biochemical nor genetic studies are not supporting this hypothesis. The best way to expand on this research would be to isolate and serotype more strains, possibly from other parts of the Mediterranean Basin, to see which characteristics are shared by certain serotypes. Furthermore, using long range high throughput sequencing in combination with Illumina sequencing would allow for the assembly of high quality genomes with high contiguity without the use of reference mapping, allowing for comparisons of most of the data, and leading to detection of more structural differences in the genome. The future directions of this research are certainly promising, as there are interesting questions still left to explore.

## Methods

### Examined strains

The bacteria compared in this study were isolated from ESB originating from the Eastern Adriatic Sea. Two strains, designated 72/16 and 150/16, were isolated during a study of the gut microbiome of the European sea bass during winter months^49^. The strain designated as 94/17 was isolated in the Laboratory for Fish Pathology of the Croatian Veterinary Institute from a diseased ESB from a Croatian farm in the central Eastern Adriatic in late summer 2017. Fish showed uncoordinated swimming, corneal opacity and haemorrhages around the mouth, on the opercula and the bases of the fins. Samples of eyes, anterior kidneys, hearts and spleens were plated on both Trypticase soy agar supplemented with 2% NaCl and Marine agar (MA) and cultured overnight at 25 °C. The obtained colonies were purified by streaking and re-streaking on fresh MA plates.

### Determination of bacterial species

Genomic DNA was extracted from bacterial cultures using the NucleoSpin Microbial DNA kit (Macherey Nagel, Germany) according to the manufacturer’s instructions. To determine the genus of the isolated bacteria, we specifically amplified 16S rRNA gene using 27FYM (5’–AGA GTT TGA TYM TGG CTC AG–3’)^50^ and 1492R (5’– TAC GGY TAC CTT GTT ACG ACT T –3’) primers^51^. Reactions were performed using GoTaq G2 Hot Start Colorless Master Mix (Promega, USA) on ProFlex PCR System (Applied Biosystems, USA). The following temperature profile was used: enzyme activation for 2 min at 95 °C, followed by 35 cycles of denaturation for 1 min at 94 °C, annealing for 30 s at 49 °C and elongation for 2 min at 72 °C, ending with the final elongation step for 5 min at 72 °C. All reactions were performed in a total volume of 20 μL, with 100 ng DNA as measured on a DS-11 Series Spectrophotometer (DeNovix, USA) and the final concentration of primers was 0.5 μM. To check for the presence of an amplified product, electrophoresis was performed on the QIAxcel Advanced System (Qiagen, Germany) using the QIAxcel DNA Screening Kit. Positive reactions were submitted for sequencing to Macrogen Europe (Amsterdam, Netherlands). Sequence alignments and BLAST were performed using Geneious Prime 2019.2. Once the genus was identified as *Vibrio*, we performed a second PCR using the same DNA samples, to test whether the bacteria belong to the species *V. harveyi*. For this purpose, we specifically amplified the *toxR* gene using primers toxRF1 (5’- GAA GCA GCA CTC ACC GAT -3’) and toxRR1 (5’- GGT GAA GAC TCA TCA GCA -3’)^53^. Conditions used for the second PCR were the same as those used in the first PCR, except the annealing temperature was 55 °C, and the duration of the elongation step in each cycle was 1 min. Electrophoresis was performed as above, and all positive samples were submitted to Macrogen Europe for sequencing.

### Serology

Rabbit polyclonal antisera against formalin-killed cells of strains belonging to dominant serotypes were prepared as previously described^35^. Then, slide agglutination with whole cell suspensions containing 10^8^ colony forming units (cfu)/mL and O-antigens in PBS were performed according to the procedure of Fouz and Amaro^36^.

### Determination of biochemical characteristics

Several tests were performed to compare the biochemical and physiological characteristics of the studied strains. Strains were grown on MA to examine colony morphology and in the selective medium TCBS agar to test their ability to ferment saccharose. Gram-stained smears of bacterial colonies were examined under a reverse microscope to differentiate Gram-positive and Gram-negative bacteria. Motility was determined using an API M Medium (BioMerieux, France), while the glucose oxidation-fermentation test was performed using an OF Medium (BioMerieux, France), both according to the manufacturer's instructions. To test for the presence of indophenol oxidase, bacterial colonies were rubbed onto filter paper treated with Oxidase Reagent Droppers (Becton, Dickinson and Company, USA), and the colour change was observed. The production of catalase was tested by observing the production of oxygen bubbles after the addition of 3% solution of hydrogen peroxide to the bacterial colony on the glass slide. To determine the type of haemolysis, bacterial colonies were grown on blood agar supplemented with 1.5% NaCl for 24 h at 23 ± 2 °C. Using the API 20E commercial test kit (BioMerieux, France) according to the manufacturer’s instructions, strains were examined for 21 biochemical properties: the presence of β-galactosidase, arginine dihydrolase, lysine decarboxylase, ornithine decarboxylase, urease, tryptophan deaminase, tryptophanase and gelatinase, utilization of citrate, production of hydrogen sulphide, fermentation of glucose, mannose, inositol, sorbitol, rhamnose, sucrose, melibiose, amygdalin and arabinose, reduction of nitrates to nitrites and Voges-Proskauer test (detection of acetoin). Salt tolerance of the strains was determined in peptone water with the addition of 0.5%, 3%, 6% and 10% NaCl. The identification was performed based on the reference characteristics described by Austin & Austin^39^ and Pretto^40^.

### Antimicrobial susceptibility testing

Antimicrobial susceptibility of the studied strains was determined by disc diffusion method^50^, on Mueller–Hinton agar (Difco, USA) supplemented with 1.5% NaCl using the following antibiotic discs: ampicillin 10 μg (Becton, Dickinson and Company (BD), USA), ceftazidime 30 μg (BD, USA), chloramphenicol 30 µg (BD, USA), enrofloxacin 5 μg (BD, USA), florfenicol 30 μg (Oxoid, UK), gentamicin 10 μg (BD, USA), meropenem 10 µg (BD, USA), novobiocin 5 µg (BD, USA), O129 10 µg (Oxoid, UK), oxolinic acid 2 µg (Biolab, Hungary), oxytetracycline 30 µg (BD, USA), sulfamethoxazole 300 µg (Biolab, Hungary) and trimethoprim/sulfadiazine 1.25/23.75 µg (BD, USA). Three criteria were used in selection of antimicrobial substances^44^; (i) agents used in therapy of aquatic vibriosis namely amoxicillin, oxolinic acid, flumequine, enrofloxacin, oxytetraciycline, florfenicol and trimethoprim/sulfametoxazole; (ii)agents used in therapy of humans infected with *Vibrio* sp. (quinolones, the third generation cephalosporins and the tetracyclines)^54^; (iii) miscelaneous criteria which includes chloramphenicol, prohibited substance that could indicate illegal use, Ceftazidime as an effective prescreen for isolates containing extended-spectrum β-lactamase-/ AmpC β-lactamase resistance mechanisms as major concern for human pathogens, meropenem is included to screen for isolates producing carbapenems^55^ and sulfamethoxazole was included to facilitate the detection of isolates possessing *sul* genes reported as occurring with a high frequency in some aquatic environments.

### Whole genome sequencing

Library preparation for high throughput sequencing was performed using NexteraXT Library Prep Kit (Illumina, USA) with two modifications to the manufacturer’s protocol: for input DNA we used two nanograms instead of one, and PCR elongation time was increased to one minute. DNA quantification and library preparation were carried out on a Hamilton Microlab STAR automated liquid handling system. Pooled libraries were quantified using the Kapa Biosystems Library Quantification Kit for Illumina on a Roche light cycler 96 qPCR machine. Libraries were sequenced on an Illumina MiSeq System using a 250 bp paired end protocol.

### Sequence analysis

Reads obtained by sequencing were adapter trimmed using Trimmomatic v0.30^56^ with a sliding window quality cut-off of Q15. After trimming, reads were mapped to the genome sequence of the *V*. *harveyi* strain ATCC 33,843^42^ in Geneious Prime 2020.1.2 using Geneious mapper. For mapping, we used medium–low sensitivity setting and fine tuning with 10 iterations. Annotations were transferred from the reference genome to consensus sequences using the following settings: 90% similarity cut-off, cost matrix 65% similarity (5.0/− 4.0) and only transfer best match. After mapping, the variants were called and annotated using the Find Variations/SNPs analysis in Geneious Prime 2020.1.2 with the following settings: minimum coverage 10, minimum variant frequency 0.5, maximum variant *P*-value 10^–6^, minimum strand-bias *P*-value 10^–5^ when exceeding 65% bias, find only nonsynonymous variants, analyse the effect of variants on translations using bacterial genetic code, merge adjacent variations and use separate annotations for each variant at a position. To align the consensus genomes we used the progressive Mauve algorithm with default settings^57^. Consensus genome sequences were exported in a FASTA format file which was used as an input file for the VFanalyzer in the VFDB^34^ and for RGI analysis in CARD^39^.

## Supplementary Information

Supplementary Information 1.

Supplementary Information 2.
