# Author Correction: Biochemical and molecular characterization of three serologically different *Vibrio harveyi* strains isolated from farmed *Dicentrarchus labrax* from the Adriatic Sea

**DOI:** 10.1038/s41598-022-24693-6

**Published:** 2022-12-01

**Authors:** Željko Pavlinec, Ivana Giovanna Zupičić, Dražen Oraić, Ivana Lojkić, Belén Fouz, Snježana Zrnčić

**Affiliations:** 1 Croatian Veterinary Institute, 10000 Zagreb, Croatia; 2 University of Valencia, 46010 Valencia, Spain

Correction to: *Scientific Reports* 10.1038/s41598-022-10720-z, published online 04 May 2022

The Acknowledgements section in the original version of this Article was incomplete.

“Genome sequencing was provided by MicrobesNG (www.microbesng.uk) which is supported by the BBSRC (grant number BB/L024209/1). We thank our colleagues Ivona Mladineo and Slaven Jozić for sharing their strains.”

now reads:

“Genome sequencing was provided by MicrobesNG (www.microbesng.uk) which is supported by the BBSRC (grant number BB/L024209/1). We thank our colleagues Ivona Mladineo and Slaven Jozić for sharing their strains. The study was also supported by the Spanish grant THINKINAZUL/2021/027 from MCIN (Ministerio de Ciencia e Innovación de España) with funding from European Union NextGeneration EU (PRTR-C17.I1) and GV (Generalitat Valenciana)."

The original Article has been corrected.

